# High Level of GMFG Correlated to Poor Clinical Outcome and Promoted Cell Migration and Invasion through EMT Pathway in Triple-Negative Breast Cancer

**DOI:** 10.3390/genes14061157

**Published:** 2023-05-26

**Authors:** Yonglin Zhao, Xing Wei, Jia Li, Yan Diao, Changyou Shan, Weimiao Li, Shuqun Zhang, Fei Wu

**Affiliations:** 1 Department of Oncology, The Second Affiliated Hospital of Xi’an Jiaotong University, Xi’an 710004, China; zhaoyonglinlin@163.com (Y.Z.);; 2 Department of Gynaecology and Obstetrics, The Second Affiliated Hospital of Xi’an Jiaotong University, Xi’an 710004, China; 3 Key Laboratory of Environment and Genes Related to Diseases, Xi’an Jiaotong University, Ministry of Education of China, Xi’an 710061, China

**Keywords:** glia maturation factor γ, triple-negative breast cancer, prognosis, cell migration, epithelial–mesenchymal transition

## Abstract

Triple-negative breast cancer (TNBC) has a very poor prognosis due to the disease’s lack of established targeted treatment options. Glia maturation factor γ (GMFG), a novel ADF/cofilin superfamily protein, has been reported to be differentially expressed in tumors, but its expression level in TNBC remains unknown. The question of whether GMFG correlates with the TNBC prognosis is also unclear. In this study, data from the Cancer Genome Atlas (TCGA), Clinical Proteomic Tumor Analysis Consortium (CPTAC), Human Protein Atlas (HPA), and Genotype-Tissue Expression (GTEx) databases were used to analyze the expression of GMFG in pan-cancer and the correlation between clinical factors. Gene Set Cancer Analysis (GSCA) and Gene Set Enrichment Analysis (GSEA) were also used to analyze the functional differences between the different expression levels and predict the downstream pathways. GMFG expression in breast cancer tissues, and its related biological functions, were further analyzed by immunohistochemistry (IHC), immunoblotting, RNAi, and function assay; we found that TNBC has a high expression of GMFG, and this higher expression was correlated with a poorer prognosis in TCGA and collected specimens of the TNBC. GMFG was also related to TNBC patients’ clinicopathological data, especially those with histological grade and axillary lymph node metastasis. In vitro, GMFG siRNA inhibited cell migration and invasion through the EMT pathway. The above data indicate that high expression of GMFG in TNBC is related to malignancy and that GMFG could be a biomarker for the detection of TNBC metastasis.

## 1. Introduction

Breast cancer is the most common malignancy worldwide [1]. In recent years, early detection and treatment have become the main factors responsible for increasing the survival rate of breast cancer patients. However, invasion and metastasis are still principal causes of death and a major challenge in the treatment of breast cancer. Breast cancer can be divided into four subtypes according to its different pathological features, including Luminal A, Luminal B, human epidermal growth factor receptor-2 (Her-2) neu, and basal-like subtypes [2]. Triple-negative breast cancer (TNBC) occurs when a patient is negative for estrogen receptor (ER), progesterone receptor (PR), and Her-2 [3], and this variant represents approximately 15–20% of all breast cancers. Treatment options for TNBC are limited, and chemotherapy for TNBC patients remains a poor choice because of the lack of effective diagnostic and prognostic markers.

Glia maturation factors (GMFs), a member of the ADF/cofilin superfamily of proteins, have been reported to be an important regulator for actin cytoskeleton reorganization [4]. Moreover, glia maturation factor γ (GMFG), a subtype of GMF, is a 17-kDa protein that can be detected in healthy individuals [5]. Researchers have reported that GMFG is enriched in various organs in humans, including the thymus, colon, and spleen [6]. GMFG has also been shown to regulate the directional migration of monocytes, neutrophils, and T-lymphocytes, as well as to modulate toll-like receptor 4 (TLR4) signaling [7,8,9,10]. Most importantly, however, GMFG has also been reported to be closely correlated to the tumor’s prognosis. High GMFG is related to a poor prognosis in colorectal cancer and epithelial ovarian cancer [11,12]. However, the role of GMFG in breast cancer, especially in TNBC, remains unknown and demands further investigation.

In this study, we assessed the GMFG expression and prognosis of patients using TCGA, and we also evaluated the expression of GMFG in breast cancer subtypes. We found that GMFG is mainly expressed in TNBC specimens and cell lines. Then, we analyzed patient clinicopathological data, especially the histological grade and axillary lymph node metastasis. Survival analysis showed that GMFG is relevant to poorer outcomes. Furthermore, we investigated the roles of GMFG in TNBC cell migration and invasion. Knocking down the expression of GMFG by siRNA significantly reduced the migration and invasion ability of MDA-MB-231 cells and inhibited TGF-β-induced epithelial–mesenchymal transition (EMT). This demonstrates that GMFG plays an oncogene role in the progression of TNBC and provides new insight that GMFG may be a new predictor for TNBC patients.

## 2. Materials and Methods

### 2.1. Data Collection

Through TCGA, we obtained mRNA expression, copy number changes, and clinical information for more than 30 cancers. mRNA expression data for normal tissue sites were obtained from GTEx at the UC Santa Cruz Xena Portal (xena.ucsc.edu (accessed on 8 December 2021)).

### 2.2. GMFG Expression Profiles in Human Cancers

We used the Wilcoxon rank sum test to analyze the expression levels of GMFG in 33 types of cancers. TIMER (http://timer.cistrome.org/ (accessed on 8 December 2021)) is a powerful online interactive website for analyzing tumor immune and gene expression [13], and we used it to analyze the expression of GMFG in various cancer types. The protein expression levels of GMFG were analyzed with the CPTAC database in different cancers (http://ualcan.path.uab.edu/analysis-prot.html (accessed on 8 December 2021)).

### 2.3. GMFG Expression Profiles in Breast Cancer

We further explored the mRNA expression level of GMFG in the UALCA database and Human Protein Atlas (HPA) (https://proteinatlas.org/ (accessed on 8 December 2021)) [14,15]. The HPA database provided the IHC of GMFG protein expression in certain tissues, and the gene expression dataset for GMFG in human breast cancer cells was obtained from the Gene expression-based Outcome for Breast Cancer Online (GOBO) database. All data were obtained using the default settings and protocols specified by the authors.

### 2.4. The Relationship between GMFG Expression and Breast Cancer Clinicopathological Features

Bc-GenExMiner (http://bcgenex.ico.unicancer.fr/BC-GEM/GEM-Accueil.php (accessed on 8 December 2021)) is an online interactive tool for studying BRCA using data from TCGA, METABRIC, and SCAN-B [16]. We used this tool to explore the GMFG expression in different breast cancer subtypes.

### 2.5. Function Enrichment Analysis

The GSCA database (http://bioinfo.life.hust.edu.cn/GSCA/#/immune (accessed on 8 December 2021)) was used to estimate the differences in gene expression between activating and inhibiting pathway groups, and we quantified these differences using median pathway scores. We also used GSEA to perform enrichment analysis in order to reveal differences between the different expression level groups of GMFG. To do this, we downloaded the annotated gene set “c2.cp.kegg.v7.4.symbols.gmt” from the Molecular Signatures Database and set the significance levels at FDR < 0.25 and *p* < 0.05 (for all the other experiments), as researchers have done previously [17,18].

### 2.6. Patients, Breast Cancer Tissues, and Breast Cancer Cell Lines

We collected specimens from 253 breast cancer patients who were undergoing surgery, including Luminal A (*n* = 28), Luminal B (Her 2−) (*n* = 27), Luminal B (Her 2+) (*n* = 34), Her-2+ (*n* = 11), and TNBC (*n* = 153), which were retrospectively collected and investigated by immunohistochemistry and survival prognostic analysis. According to the National Comprehensive Cancer Network (NCCN) guidelines, a tumor recurrence that took place within 7 months after chemotherapy and radiotherapy was defined as recurrence. After this, we collected the clinicopathological and follow-up data of the patients. At the time of the last follow-up, we observed that 111 of the 153 (72.5%) TNBC patients had survived. All the specimens were obtained from the Department of Oncology, The Second Affiliated Hospital of Xi’an Jiaotong University, China, and ethical approval was granted by the Ethical Committee of Xi’an Jiaotong University.

All the breast cancer cell lines reported in this study were readily available in our laboratory, including MDA-MB-231, MDA-MB-435, ZR-75B, MDA-MB-436, BT-549, and MCF-7. All the cell lines were cultured in suitable media, such as DMEM and 1640, with the addition of 10% FBS and 1% ps, and then incubated.

### 2.7. siRNA and Transfection

We used GMFG siRNA (Thermo Fisher Scientific, Waltham, MA, USA, 137798) to knockdown GMFG expression using Lipofectamine 2000 (Invivogen, San Diego, CA, USA) according to the protocol of the manufacturer and a previous study [19].

### 2.8. Immunoblotting

Tissue samples and cells were harvested with RIPA buffer (Wolsen, Xi’an China) and quantitated with BCA (Pioneer biology, Xi’an China). The protein sample went through the following steps: separation, transfer, blocking, and incubation with a primary antibody and secondary antibodies to detect the protein’s expression. The antibodies used were GMFG (Cat No. 13625-1-AP), E-cadherin (Cat No. 20874-1-AP), N-cadherin (Cat No. 22018-1-AP), vimentin (Cat No. 10366-1-AP), snail (Cat No. 13099-1-AP), and β-actin (Cat No. 66009-1-Ig) (all 1:1000). All the primary antibodies were purchased from Proteintech (Wuhan China). The secondary HRP-conjugated anti-rabbit (GTX213110-01) or anti-mouse (GTX213111-01) antibodies were purchased from GENTEXT, Inc. (Carbondale, PA, USA) (1:5000).

### 2.9. Wound Healing Assay

On day 1, we planted the breast cells into 6-well plates, then transfected them on day 2. When the cells had grown to 80% density in the well plate, we created a linear scratch using a 10 µL disposable pipette tip, then measured the wound closure at 24, 36, and 48 h after wounding.

### 2.10. Transwell Migration and Invasion Assay

Transwell chambers were used to study cellular migration and invasion. We used Matrigel to detect the invasion ability of the cells as follows. First, we planted cells in 24-well plates and transfected them on day 2. Then, 24 h later, we filled the upper chamber with 200 μL of serum-free medium and the bottom of the plate with 600 μL of 10% FBS medium. We then harvested the cells from the 24-well plates and planted 2.0 × 10^4^ cells in the upper chamber. Finally, we observed the ability of the cells to cross the membrane from the upper chamber to the lower chamber.

### 2.11. Immunohistochemistry

For our immunohistochemistry analysis, slides were incubated with a primary antibody against GMFG (Abcam, diluted 1/500), and then an IHC kit (ZSGB-BIO, PV-9002) was used. These samples were then observed under a 40× microscope. Determination of the immunohistochemical scores (IHS) using Image-Pro Plus 6.0 software then proceeded as follows. (1) The numbers are scored on a 0–4 scale, with 0 for no staining; 1 for 1–10% of cells stained; 2 for 11–50%; 3 for 51–80%; and 4 for 81–100%. (2) Staining intensity was rated on a scale of 0–3, with 0 = negative, 1 = weak, 2 = moderate, and 3 = strong. The product of the above two scores was taken as the score of the field of view. Ideally, the final score ranged from 0 to 12. A score of 0–4 was defined as “low expression”, a score of 5–8 as “moderate expression”, and a score of 9–12 as “high expression”. All the scores were confirmed by two independent pathologists according to the IHS standards [20,21,22 and 23].

### 2.12. Statistical Analysis

All the statistical analysis was performed using SPSS 20.0 statistical software (SPSS, Chicago, IL, USA). The primary variable of interest was survival, so we used the Kaplan–Meier technique to plot the survival curves of the patients. Prior to analysis, all the data were expressed as mean ± SD and statistically analyzed using one-way ANOVA and χ2 tests. For all the tests, a *p* < 0.05 indicated a statistically significant result.

## 3. Results

### 3.1. GMFG Is Differentially Expressed in Human Cancers

**Figure 1:**
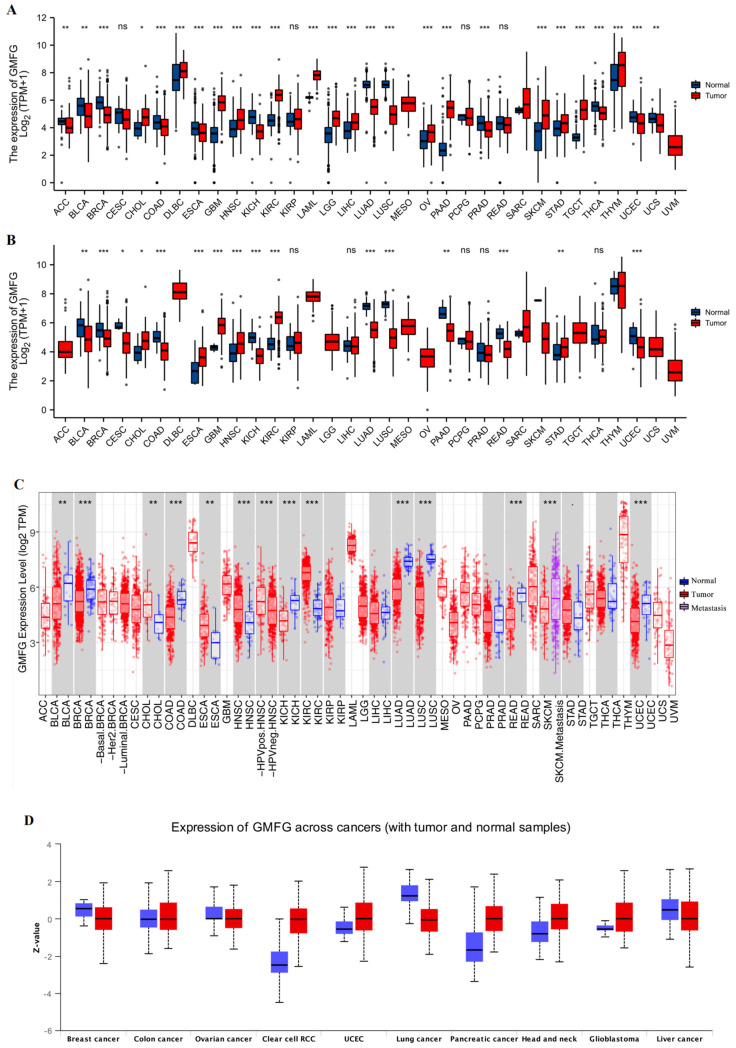
The mRNA expression of GMFG in various cancers according to The Cancer Genome Atlas (TCGA), Genotype-Tissue Expression (GTEx), and CPTAC. (**A**) GMFG expression in pan-cancer relied on TCGA. (**B**) GMFG expression in pan-cancer based on TCGA, and the normal samples were enriched with data from the GTEx database. (Wilcoxon rank sum test, * *p* < 0.05, ** *p* < 0.01, *** *p* < 0.001, ns: no significance. N: Normal tissue; T: Tumor tissue.) (**C**) GMFG expression in pan-cancer relied on TIMER. (**D**) GMFG protein expression in pan-cancer relied on CPTAC.

We used TCGA to analyze the expression of GMFG in tumor and normal control samples in various tissue types. GMFG expression was upregulated in tumor tissues in several cancers, including cholangiocellular carcinoma (CHOL), esophageal carcinoma (ESCA), glioblastoma (GBM), head and neck squamous cell carcinoma (HNSC), kidney renal clear cell carcinoma (KIRC), and stomach adenocarcinoma (STAD) (Figure 1A), and was downregulated in bladder urothelial carcinoma (BLCA), breast invasive carcinoma (BRCA), cervical squamous cell carcinoma and endocervical adenocarcinoma (CESC), colon adenocarcinoma (COAD), kidney chromophobe (KICH), lung adenocarcinoma (LUAD), lung squamous cell carcinoma (LUSC), pancreatic adenocarcinoma (PAAD), rectum adenocarcinoma (READ), and uterine corpus endometrial carcinoma (UCEC) (Figure 1A). Considering that several cancers lacked moderate corresponding normal tissue controls in the TCGA database, which placed restrictions on the statistical credibility of these results, we further combined the TCGA and GTEx databases to explore the GMFG expression in a more robust manner and found that high GMFG expression was observed in 15 tumors: CHOL, lymphoid neoplasm diffuse large b-cell lymphoma (DLBC), GBM, HNSC, KIRC, acute myeloid leukemia (LAML), brain low-grade glioma (LGG), liver hepatocellular carcinoma (LIHC), ovarian serous cystadenocarcinoma (OV), PAAD, skin cutaneous melanoma (SKCM), testicular germ cell tumors (TGCT), thymoma (THYM), and STAD. Low GMFG expression was observed in 13 tumors: adrenocortical carcinoma (ACC), BLCA, BRCA, COAD, ESCA, KICH, LUAD, LUSC, prostate adenocarcinoma (PRAD), READ, thyroid carcinoma (THCA), uterine carcinosarcoma (UCS), and UCEC (Figure 1B). The results from the TIMER database were consistent with the above findings, as well (Figure 1C). The protein expression levels of GMFG in various tumors are shown in Figure 1D. High GMFG protein expression was observed in clear cell renal cell carcinoma (ccRCC), UCEC, PAAD, and HNSC, and low expression in breast, lung cancer, and liver cancer. In summary, we found that GMFG is differentially expressed in human cancers.

### 3.2. GMFG Is Differentially Expressed in Different Types of Breast Cancer and Significantly So in the Basal-like Subtype

**Figure 2:**
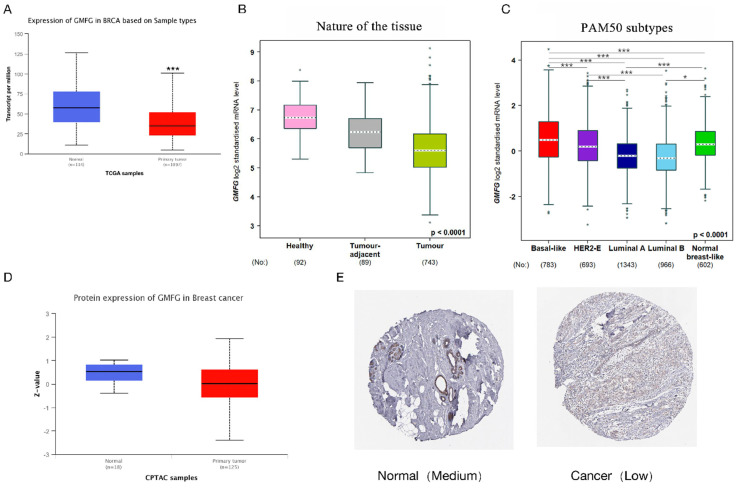

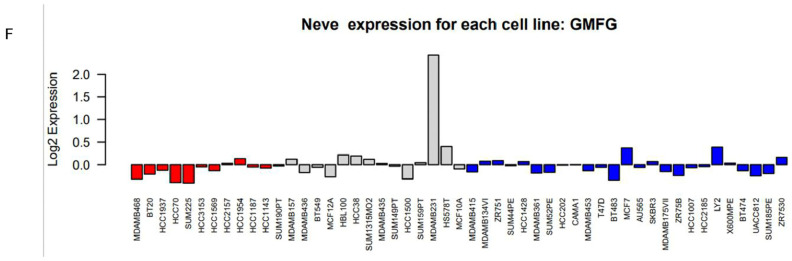
GMFG expression profiles in breast cancer. (**A**) The GMFG mRNA expression levels between breast cancer and normal tissue. (**B**) The GMFG mRNA expression levels among breast cancer, tumor-adjacent tissue, and healthy tissue. (**C**) The GMFG mRNA expression levels among PAM50 subtypes. (* *p* < 0.05, *** *p* < 0.001). (**D**) The GMFG protein expression levels between breast cancer and normal tissue. (**E**) GMFG protein levels were medium in normal tissue and low in tumor cells. (**F**) Expression of GMFG mRNA in different subtypes of breast cancer cells (basal-A, red, basal-B, gray, luminal, blue) based on expression values derived from the GOBO database.

Since breast cancer is the most commonly diagnosed malignant tumor, we further explored the expression pattern of GMFG in breast cancer, specifically. Figure 2A shows that the mRNA expression level of GMFG in breast cancer is lower than that in normal tissue, and Figure 2B shows that the mRNA expression level of GMFG is the lowest in breast cancer compared to the tumor-adjacent tissues and healthy tissues. We further compared the mRNA expression level of GMFG in PAM50 subtypes of breast cancer and found that the basal-like subtype has significantly higher expression levels than the other four subtypes (Figure 2C). Figure 2D shows that the protein expression level of GMFG in breast cancer is lower than in normal tissue. Next, we further examined the GMFG protein expression profile in breast cancer using the HPA database and found that the staining of GMFG protein in breast cancer is low, but moderate in normal tissue (Figure 2E). Finally, we further investigated the expression level of GMFG in different breast cancer cell lines using the GOBO database. Basal-B breast cancer cells show high GMFG expression, but basal-A and luminal breast cancer cells express low or undetectable levels (Figure 2F).

### 3.3. Correlation between GMFG Expression and Clinicopathological Parameters of Breast Cancer Patients

**Figure 3:**
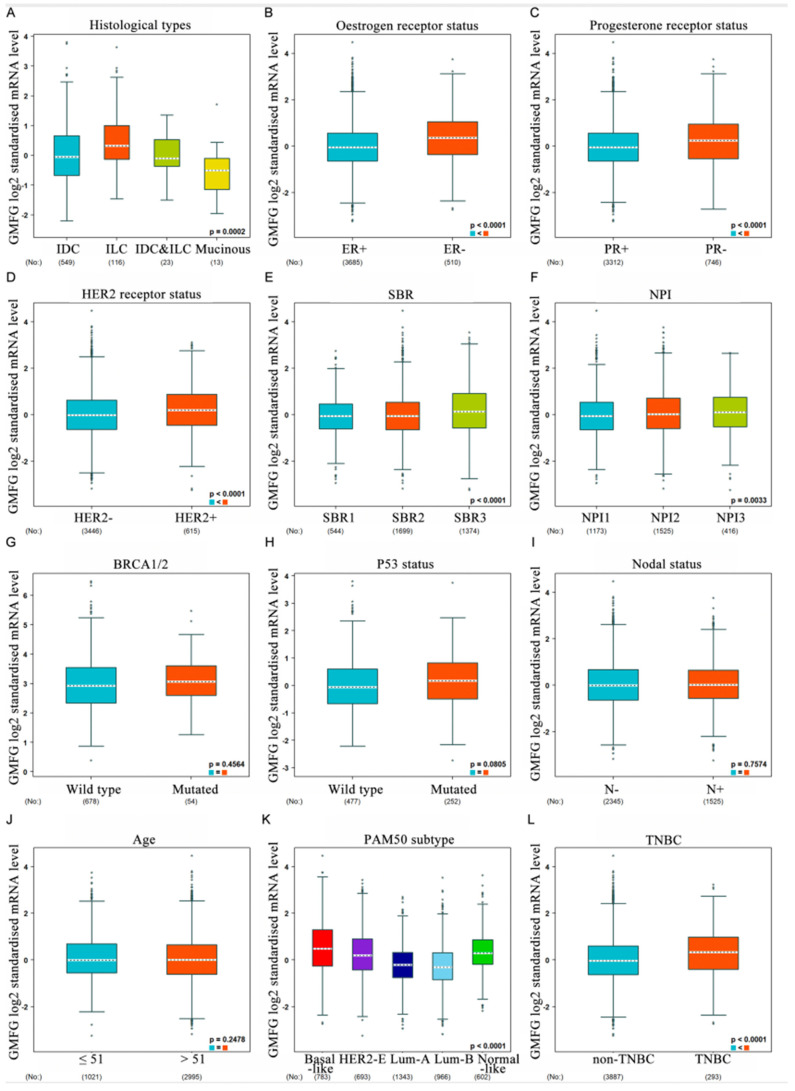
The violin plot illustrates the relationship of GMFG expression with clinical indicators using bc-GenExMiner. Data include histological types (**A**), ER (**B**), PR (**C**), HER-2 (**D**), SBR (**E**), NPI (**F**), BRCA1/2 status (**G**), TP53 (**H**), nodal status (**I**), age (**J**),TNBC status (**K**), and basal-like status (**L**), (* *p* < 0.05).

We used bc-GenExMiner datasets to assess whether GMFG expression is correlated with breast cancer patients’ clinicopathological parameters. Figure 3A shows that there is a dependency between GMFG expression and the histological data of patients, and GMFG expression is highest in invasive ductal carcinoma (IDC) (Figure 3A). For mRNA levels, the expression of GMFG is higher in ER− than in ER+ patients (ER−  >  ER+, *p*  <  0.0001) (Figure 3B), and lower in the PR− group than in the PR+  group (PR+  >  PR−, *p* = 0.01) (Figure 3C). In addition, the expression of GMFG is upregulated in the HER2+ group vs. in the HER2− group (HER2+  >  HER2−, *p* < 0.001) (Figure 3D). Figure 3E shows that SBR levels are related to GMFG transcript levels (SBR3  >  SBR2  >  SBR1, *p*  <  0.0001), and analysis by the Nottingham prognostic index (NPI) criteria showed that increased SBR levels are associated with elevated GMFG transcript levels (NPI3  >  NPI2  >  NPI1, *p*  <  0.0001) (Figure 3F). There was no correlation between GMFG levels and BRCA1/2 status, TP53 status, lymph node status, or histological type, however (Figure 3G–J). Additionally, GMFG is significantly upregulated in basal-like subtypes compared to non-basal-like subtypes (Figure 3K), and this is true of TNBC in particular (Figure 3L).

### 3.4. Function Enrichment Analysis

**Figure 4:**
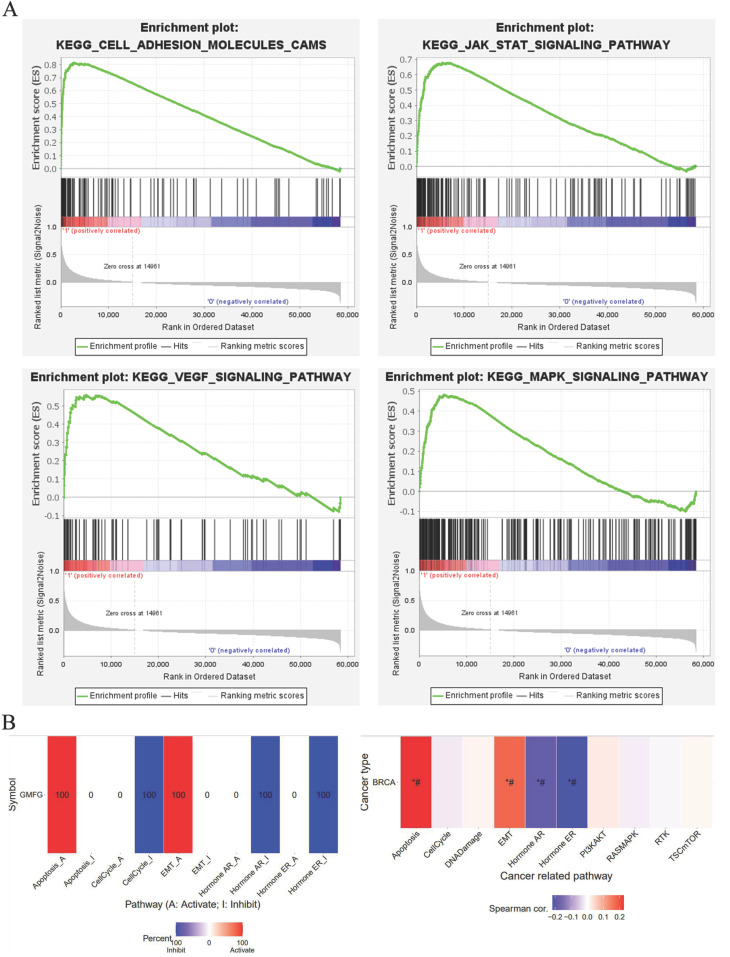
Function analysis of GMFG. (**A**) GSEA revealed the four pathways were enriched in the high GMFG expression group. NES, normalized ES; FDR, false discovery rate. (**B**) GSCA indicated the potential effect of GMFG on significant cancer-related pathways.

All of the above results show that GMFG is specifically highly expressed in basal-like breast cancer. What is the possible mechanism behind this? To help answer this question, we performed functional enrichment analysis as a preliminary exploration. The results of GSEA testing showed that there were 61 pathways enriched in the high-expression GMFG group, which were mainly associated with cancer, cell cycle, DNA repair, and immune function: “KEGG_CELL_ADHESION_MOLECULES_CAMS”, “KEGG_JAK_STAT_SIGNALING_PATHWAY”, “KEGG_VEGF_SIGNALING_PATHWAY”, and “KEGG_MAPK_SIGNALING_PATHWAY” (Figure 4A). Figure 4B illustrates that GMFG’s mRNA expression has potential activation effects on apoptosis and EMT pathways in breast cancer but potential inhibitory effects on the cell cycle, hormone ER, and PR pathways. We didn’t find any relationship between GMFG mRNA expression and the PI3K/AKT, RTK, or TSC/mTOR pathways in breast cancer (Figure 4B). These results suggest the potential carcinogenic effects of GMDG in TNBC.

### 3.5. GMFG Is Significantly Upregulated in TNBC Samples and Is Related to Poor Prognosis for TNBC Patients

**Figure 5:**
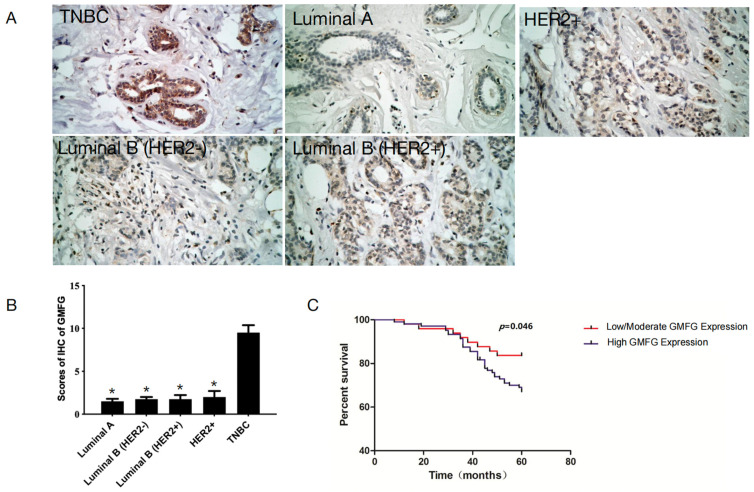
Evaluation of GMFG expression in different subtypes of breast cancer. (**A**) Staining of GMFG in Luminal A, Luminal B (Her 2−), Luminal B (Her 2+), and Her-2+ breast cancer types, respectively, by immunohistochemistry. (**B**) The bar graphs show the IHS of GMFG expression in different subtypes of breast cancer. Values are presented as the mean ± SD (compared with TNBC group, * *p* < 0.05). (**C**) Kaplan–Meier curves for overall survival of TNBC patients classified by their GMFG staining intensity.

To examine GMFG expression in all the subtypes of breast cancer tissues, we used IHC to detect the protein levels of GMFG in 31 Luminal A, 28 Luminal B (Her 2−), 27 Luminal B (Her 2+), 34 Her-2+, and 37 TNBC primary breast cancer tissues. In all cases, higher levels of GMFG were expressed in the TNBC samples but low or undetectable levels were found in the Luminal A, Luminal B (Her 2−), Luminal B (Her 2+), and Her-2+ breast cancer subtypes (*p* < 0.05) (Figure 5A,B). These results indicate that GMFG is significantly over-expressed in TNBC.

We then collected more samples to explore if GMFG expression was related to the clinicopathological parameters of TNBC patients, and the sample size was increased to the full 153. We divided the TNBC patients into low/moderate and high expression groups and then analyzed the correlations of the various parameters to the expression levels (Table 1). The results show that GMFG expression is significantly correlated with histological grade (*p* = 0.033) and axillary lymph node metastasis (*p* = 0.027) but had no significant correlation with patient age, tumor size, and/or menopause.

Next, we asked, can GMFG reflect the prognosis of TNBC patients? To answer this question, we used a Kaplan–Meier analysis. The five-year OS of high-GMFG-expressing patients was 67.31% (70/104), and 83.67% (41/49) for patients with low/moderate GMFG expression. The TNBC patients with highly expressed GMFG showed shorter overall survival (*p* = 0.043, Figure 5C). Therefore, we speculate that the expression of this gene is correlated with a poorer prognosis.

### 3.6. GMFG Promotes TNBC Cell Migration and Invasion by Regulating the TGF-β-Mediated EMT Signaling Pathway

**Figure 6:**
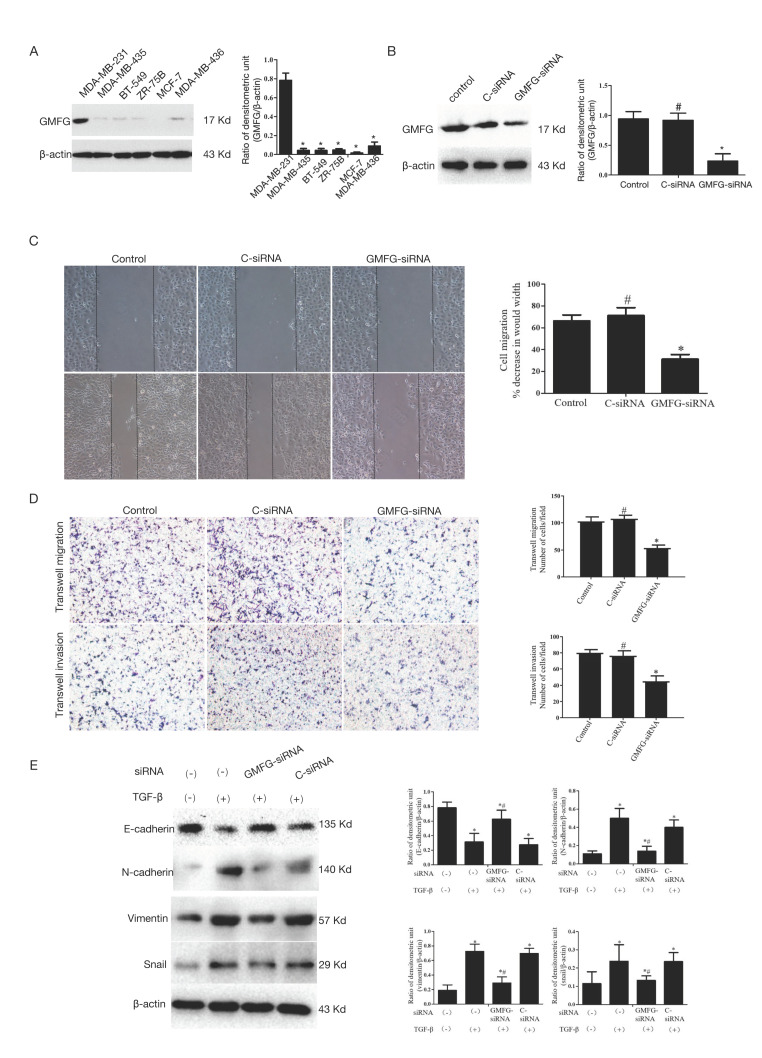
GMFG promotes TNBC cell migration and invasion. (**A**) GMFG protein-level measurement in breast cancer cell lines by immunoblotting analysis. (**B**) GMFG siRNA reduced the expression of GMFG assessed by Western blot. (**C**) Knockdown of GMFG inhibited wound-scratch healing (**D**) Knockdown of GMFG reduced cell migration and invasion. (**E**) Effect of GMFG siRNA on E-cadherin, N-cadherin, vimentin, and snail expression in TGF-β stimulated MDA-MB-231 cells. Values are presented as the mean ± SD (* *p* < 0.05, # *p* > 0.05).

GMFG expression was tested on breast cancer cell lines including three basal-like (BT-549, MDA-MB-231, MDA-MB-436), two luminal-like (MCF-7, ZR-75B), and one Her-2 (MDA-MB-435). Significantly, GMFG was distinctly highly expressed in MDA-MB-231 cells (*p* < 0.05, Figure 6A). Thus, we selected MDA-MB-231 for further investigation in vitro.

The expression of GMFG in these samples was obviously reduced by GMFG siRNA assessed by Western blotting (*p* < 0.05, Figure 6B). Compared to the C-siRNA group, MDA-MB-231 with a decreased expression of GMFG showed a weaker ability to migrate (*p* > 0.05, Figure 6C). The abilities to transwell migrate and invade the MDA-MB-231 cells were also suppressed by GMFG siRNA compared to the C-siRNA group (*p* < 0.05, Figure 6D).

We also investigated the effects of GMFG on the EMT of the MDA-MB-231 cells, and the results showed that MDA-MB-231 cells treated with TGF-β had lower E-cadherin expression and higher vimentin, N-cadherin, and snail expression, which is considered a hallmark of EMT (*p* < 0.05). GMFG silencing significantly increased E-cadherin but decreased N-cadherin, vimentin, and snail expression (*p* < 0.05, Figure 6E), indicating that GMFG siRNA inhibited EMT in TGF-β-induced MDA-MB-231 cells.

## 4. Discussion

Strategies for efficiently combating TNBC have yet to be developed. In an effort to rectify this, we assessed GMFG as a potential new target and a novel prognostic indicator for the treatment and diagnosis of TNBC. GMFG, a member of the ADF/cofilin superfamily of proteins, is mainly expressed in inflammatory cells and is involved in regulating the reorganization of actin in a variety of cells, including cancer cells [11,12,24]. We first found that GMFG was highly expressed in a TNBC cell line from the public repository of GeneChips (GEO, GSM2695488, GSM2695489, GSM2695490, GSM2695491, GSM2695492, and GSM2695493). However, the mechanism of abnormal GMFG expression in breast cancer cell lines remains unclear. Further investigation revealed that GMFG was specifically increased in MDA-MB-231 compared to other breast cancer cell lines, the same as in the GEO database.

In addition, we explored the relationship between GMFG expression and the pathological significance of BC patients from Northwest China and revealed that the expression of GMFG was specially increased in TNBC patients. A high expression of GMFG was also found to be associated with histological grade and axillary lymph node metastasis in these patients. Survival analysis suggested that the five-year OS was 67.31% in patients with high GMFG expression and 83.67% for low/moderate expression patients. Therefore, we speculate that the expression of this GMFG is correlated with poorer prognosis in TNBC.

Migration and invasion of cancer cells are necessary for tumor metastasis, and, in this regard, GMFG may enhance tip cell sprouting, tube formation, and the directional migration of monocytes, neutrophils, and T-lymphocytes [8,9,10]. A previous study has shown that a high GMFG expression in epithelial ovarian cancer cells exhibits stronger migration and invasion abilities [12]. Similar results have also been found in colorectal cancer cells [11]. In our study, we explored the role of GMFG in the cell migration of TNBC cells. By wound healing and chamber assays, we found that silencing GMFG reduced MDA-MB-231 cell migration and invasion ability, suggesting that high expression of GMFG in TNBC might contribute to cancer progression.

Although we found that GMFG plays a role in TNBC progression, the underlying mechanism of GMFG’s promotion of tumor metastasis still needs to be further explored. TNBC is more likely to metastasize at an early stage than other subtypes. EMT, which is thought to be a pro-metastatic event, is critical to the development of distant metastases [25,26], and the TGF-β pathway is one of the most well-studied signal transduction pathways that can induce EMT [22]. In this study, we found that MB-231 cells showed lower E-cadherin expression, and higher vimentin, N-cadherin, and snail expression after TGF-β stimulation, indicating the expanding ability of EMT. However, GMFG silencing significantly inhibited the TGF-β-induced EMT of MDA-MB-231. The mechanism of GMFG in regulating EMT in TNBC cells may be related to remodeling actin cytoskeletons. GMFG participates in actin reorganization, and, in ovarian cancer cells, GMFG over-expression can alter actin cytoskeleton organization [12]. Increasing amounts of evidence also suggest that cytoskeletons play a crucial role in the EMT process. Cytoskeletons, including actin cytoskeletons, are necessary for EMT by providing structural design and mechanical strength [23]. Cancer cells are highly likely to migrate and invade after actin cytoskeleton dynamic reorganization.

Furthermore, based on the available research, Gabrielson assessed chromosomal copy number changes in breast cancers with a ‘basal-like’ phenotype using high-resolution oligonucleotide comparative genomic hybridization arrays [22] and found that highly amplified regions (copy number changes greater than eight-fold) on 19q13.2 were particularly prominent. High-level amplification of 19q13.2 is a common genetic alteration in breast cancers [27]. The GMFG gene was also localized at 19q13.2 within the 660 kb amplicon maximum, so GMFG may be one target gene of the amplification process [28]. Thus, 19q13.2 may contain oncogenes such as GMFG that accelerate the progression of multiple tumors, especially TNBC.

## 5. Conclusions

In conclusion, we demonstrated that GMFG is exclusively over-expressed in TNBC tissues and cell lines and that elevated levels of GMFG lead to worse prognoses for TNBC patients. Further results revealed that GMFG promotes the migration and invasion of TNBC cells. The underlying mechanism of GMFG in leading to the aggressiveness and metastasis of TNBC may be related to its regulation of EMT. All of the above suggests that GMFG could, therefore, be a potential therapeutic target for TNBC patients, and the targeting of GMFG expression may be able to suppress TNBC progression.

## Figures and Tables

**Table 1:** GMFG expression and its association with the clinicopathological data from TNBC patients.

Clinicopathological Characteristics	GMFG		*p* Value
	Low/Moderate (%)	High (%)	
Age (year)			
<50	23 (30.26)	53 (69.74)	0.642
≥50	26 (33.77)	51 (66.23)	
Menopause			
Premenopausal	29 (38.67)	46 (61.33)	0.084
Postmenopausal	20 (25.64)	58 (74.36)	
Tumor size (cm)			
T1	21 (45.65)	25 (54.35)	0.120
T2	16 (26.23)	45 (73.77)	
T3	7 (29.17)	17 (70.83)	
T4	5 (22.73)	17 (77.27)	
Histological grade			
G1	19 (48.72)	20 (51.28)	0.033 *
G2	18 (27.69)	47 (72.31)	
G3	12 (24.49)	37 (75.51)	
Axillary lymph node metastasis			
Negative	32 (40.00)	48 (60.00)	0.027 *
Positive	17 (23.29)	56 (76.71)	

* *p* < 0.05. GMFG, glia maturation factor γ.

## Data Availability

All the data presented in this study are available in the article.
